# A Case of Acute Ascending Paralysis (Landry's Paralysis)

**Published:** 1890-06

**Authors:** Arthur G. Blomfield

**Affiliations:** Physician to the Devon and Exeter Hospital


### Clinical Records.

A CASE OF ACUTE ASCENDING PARALYSIS (LANDRY'S PARALYSIS). By ARTHUR G. BLOMFIELD, M.D. Aberd.; Physician to the Devon and Exeter Hospital.

THE notes of the following case, which I saw with Dr. Bothwell of Topsham, near Exeter, in December, 1889, may be of interest.

Mr. B., aged 46, has always enjoyed fairly good health; but in June, 1888, was thrown from a trap, which fell on him and loosened some of his teeth, but otherwise he had no injury to his head, and no loss of consciousness.

During the past twelve days he has had a severe attack of diarrhœa, which he attributes to catching cold.

*Two* days before I saw him in consultation with Dr. Bothwell, while getting out of bed he fell on the floor, but managed to get back into bed, assisted by his son. There had been no warnings—no weakness, no pains, no numbness in his lower limbs—before his legs suddenly "gave way" as he got out of bed. When he got back into bed, he found he was quite powerless in both legs, and he could not move them at any of the joints. Next day both arms became equally useless. There was no difficulty of breathing, and no trouble about the bladder. On the next day I saw him. He lay in bed, perfectly sensible, complaining of *nothing* beyond the complete loss of power in all four extremities. His breathing was regular, and the pulse fairly good. Temperature, 98.6°. All four extremities appeared equally paralysed. He could not execute the simplest movement with either hand, nor flex the limbs at any of the joints. The fingers were drawn somewhat into the palm. Both lower limbs were similarly paralysed. The reflexes were abolished. There was no ankle-clonus, and there were no tremors. The limbs were well nourished; there was no evidence of muscular wasting, and sensation everywhere was perfect. The abdominal and thoracic muscles were not apparently involved. There is a distinct history of *chronic* alcoholism. We gave him liquor strychninæ, ♏ iii. ter die, and applied warmth to the limbs; and ordered him to have frequent liquid nourishment, but no stimulants.

Dr. Bothwell tells me that he remained in the same state until next morning, when he got rapidly worse, with symptoms of paralysis of respiration, from paralysis of the thoracic muscles; and he died at 5 p.m., consciousness remaining to the last. The total duration, therefore, of his fatal illness was *three* days. There was, unfortunately, no *post mortem;* but, from the character of the symptoms, I think we may fairly consider the case as one of Landry's paralysis. It was of interest to note that a specimen of his urine was taken home for examination at my visit, and was found to have a sp. gr. of 1032, and to contain 21 grains of sugar to the ounce, or 4.8 per cent. Dr. Bothwell tells me that he had not complained of any symptoms suggesting diabetes; but in January, 1889, he had consulted my colleague, Mr. Bankart, for temporary loss of vision, recurring occasionally and lasting a few minutes, and usually relieved by some stimulant. He had smoked a good deal. Mr. Bankart tells me that he found on examining the fundus that the retinal veins were full, and the disc rather pale.

When I first saw the patient, I was inclined to think that possibly the case was one of wide-spread peripheral neuritis, more especially from the history given of chronic "nipping." However, many of the characteristic symptoms of this latter affection were absent, and the rapid development of the paralysis without fever, pain, or loss or alteration of sensation, and without wasting of the muscles, justifies us in concluding that the case was really one of acute ascending paralysis. Reference has recently been made to the association of diabetes and neuritis, and it would be of interest to know whether other observers have noted the association of glycosuria and acute ascending paralysis as in this case.

